# Effect of Silicon on Early Root and Shoot Phenotypes of Rice in Hydroponic and Soil Systems

**DOI:** 10.3390/plants15020176

**Published:** 2026-01-06

**Authors:** Kabita Poudel, Amit Ghimire, Minju Kwon, Mbembo Blaise wa Mbembo, Yoonha Kim

**Affiliations:** 1 Department of Food Security and Agricultural Development, Kyungpook National University, Daegu 41566, Republic of Korea; 2 Agriculture Development Section, Mechinagar Municipality, Office of the Municipal Executive, Jhapa 57207, Nepal; 3 Department of Applied Biosciences, Kyungpook National University, Daegu 41566, Republic of Korea; 4 Department of Integrative Biology, Kyungpook National University, Daegu 41566, Republic of Korea; 5 Upland Field Machinery Research Center, Kyungpook National University, Daegu 41566, Republic of Korea

**Keywords:** silicon, zeolite, root phenotypes, photosynthesis, hydroponic, soil, rice

## Abstract

Silicon (Si) application is recognized for its beneficial roles in crop growth. This study examines the effects of two forms: zeolite and sodium metasilicate (SMS), on rice under hydroponic (EP I) and soil (EP II) conditions. Four treatments were used at the early stage of rice: 4 ppm and 2 ppm of Si from zeolite, 4 ppm of Si from SMS, and a control. In EP I, only 4 ppm of SMS significantly improved root traits: total root length (36%), surface area (34%), root volume (23%), tips (46%), and forks (34%) by day seven compared to the control. Zeolite-based Si had minimal effects, except on the average diameter. However, in EP II, all Si forms enhanced root traits: total root length (50–73%), surface area (51–58%), average diameter (32–50%), root volume (54–72%), tips (29–68%) and increased shoot and root dry weights by 19–24% and 79–106%, respectively, compared to the control. In EP II, starting from the first and fifth day of treatment, the Si applied groups showed a significant increase in photosynthetic traits and vegetative indices, respectively. On the last day of treatment, particularly for 2 ppm of Si zeolite, the electron transport rate increased by 5 times, the apparent transpiration by 3 times, total conductance and stomatal conductance by around 50%, normalized difference vegetative index by 6–8%, and photochemical reflectance index by 14–33%. These results suggest that the effectiveness of Si is highly dependent on the growth medium and the type of Si, with soil enabling better Si availability, uptake, and physiological response compared to hydroponics. The superior performance of zeolite in EP II indicates its potential as a slow-release Si source that enhances root development and photosynthetic efficiency over time. Thus, it is concluded that zeolite has more potential in soil, and soluble silicon sources should be selected in hydroponics.

## 1. Introduction

The rice (*Oryza sativa* L.) is one of the most important cereal crops globally, supporting the livelihoods of billions of people, particularly in Asia and Africa [1]. Belonging to the genus *Oryza*, rice includes two cultivated species: *Oryza sativa* L., mostly grown in Asia, and *Oryza glaberrima Steud*., cultivated in Africa [2]. Nutritionally, rice is primarily composed of carbohydrates, making up approximately 80% of the grain’s dry weight [3], with proteins ranging between 6.9 and 10.2% [4], lipids between 1.7 and 3.2% [5], and dietary fiber from 1% to 3.6% [6]. It also contains small quantities of essential vitamins, including vitamin E (1–2 mg/kg), riboflavin (0.03–0.1 mg/100 g), niacin (3–5 mg/100 g), and thiamine (0.6–1.0 mg/100 g), as well as minerals such as iron (1.1–1.6 mg/100 g), zinc (1.5–2 mg/100 g), magnesium, phosphorus, and potassium [7]. Despite its nutritional value, maintaining high rice yields remains challenging due to various biotic and abiotic stresses, making the development of sustainable production strategies essential. Among different strategies, Silicon (Si) application has shown a promising effect in improving stress tolerance [8]. Si, considered as a beneficial element for many crops, constitutes about 28% of the earth’s crust by mass and is mainly found in the form of silicates and silica-containing minerals such as clay, feldspar, and quartz [9]. Among the various sources of Si, zeolite is also a commonly used agricultural mineral source. Zeolite is a porous mineral rich in silica that retains nutrients and water, releasing them slowly. Its high porosity and cation-exchange capacity enhance the soil’s physical and chemical qualities, boosting water retention and nutrient supply. Adding zeolite improves aeration and encourages root penetration and growth by increasing total soil porosity and reducing bulk density. Because of its ability to hold water within its pore structure, zeolite acts as a “reservoir,” helping soil stay moist for longer and shielding plants from drought or irregular watering irrigation [10,11,12]. Although only a small fraction of Si is taken up by plants, as monosilicic acid (H_4_SiO_4_) at concentrations ranging from 0.1 to 0.6 mM under root-zone pH < 9, its physiological role in plants is well-recognized [13]. Rice is known to accumulate large amounts of Si, up to 10% of shoot dry weight [14], which is essential for stable and high grain yields due to its ability to mitigate the effects of metal toxicity, drought, salinity, insect pests, lodging, and nutrient imbalances [15]. Agronomically, the application of Si has been associated with enhanced cell wall strength, increased photosynthetic activity, improved nutrient uptake, and greater water-use efficiency [13]. It also contributes to grain quality, yield stability, and stress resistance in rice. In rice, Si uptake and distribution are regulated by specific transporter genes. *Lsi1* facilitates Si influx into root cells, *Lsi2* mediates efflux into the xylem, and Lsi6 assists in the transfer of Si to shoot tissues. Recent findings have identified *SIET4* (Si Efflux Transporter 4) as a key gene for depositing Si in leaves, allowing rice to grow healthily under terrestrial conditions [14].

Roots play a fundamental role in plant performance, acting as anchorage for the shoot, regulating the uptake of water and essential nutrients, and serving as storage organs [16]. Root morphology and physiology indicate plant growth. Root-shoot interactions affect biomass, development, and productivity. High root activity improves nutrient uptake and photosynthesis, boosting biomass and yield [17]. Traditional root phenotyping methods, such as soil coring, excavation, and manual washing, are laborious, time-consuming, and prone to sampling bias and physical damage [18]. In contrast, image-based plant phenotyping offers a non-destructive, high-throughput alternative for quantitatively assessing both above- and belowground traits [19,20]. Imaging methods such as red, green, and blue (RGB), hyperspectral, thermal, and X-ray computer tomography (CT) imaging, enable the fast and precise assessment of plant architecture and physiological responses [21]. These techniques enable the detailed analysis of root traits, including total root length (TRL), surface area (SA), average diameter (AD), root volume (RV), and the number of tips, forks, links, and crossings. Furthermore, the integration of machine learning tools such as SegRoot and U-Net has significantly improved the resolution, speed, and accuracy of root phenotyping [21,22]. Thus, image-based root phenotyping represents a significant advancement in plant science, enabling accurate, time-resolved, and high-throughput analysis of root system architecture, which has historically been difficult to study [17]. This study builds on such advances by evaluating the comparative performance of two Si-based fertilizers, sodium metasilicate (SMS) and Si zeolite, during the early seedling growth stage of rice, aiming to inform strategies for sustainable rice production.

Apart from underground phenomics (root traits), above-ground photosynthetic traits and vegetative indices (VIs) are key for assessing plant growth, development, and stress. Photosynthetic parameters like chlorophyll content, net photosynthetic rate (Pn), stomatal conductance (gs), and transpiration efficiency directly impact a plant’s ability to convert light into biomass, affecting yield and resource efficiency [23]. VIs, including the normalized difference vegetation index (NDVI), photochemical reflectance index (PRI), anthocyanin reflectance index (ARI), and carotenoid reflectance index (CRI), provide non-destructive, rapid, and reliable insights into plant health, photosynthetic activity, and pigment dynamics [24,25]. These indices are highly sensitive to changes in chlorophyll concentration, canopy structure, and stress-induced physiological alterations, enabling early detection of nutrient deficiencies, drought stress, or disease pressure [26]. Monitoring these spectral and photosynthetic indicators enables a deeper understanding of plant responses to environmental conditions and management practices, ensuring optimal growth and sustained productivity. Moreover, integrating vegetative indices with physiological measurements improves precision in crop monitoring, facilitating early stress detection and guiding timely interventions to improve yield and resilience [27,28].

In our previous research, we focused on the use of silicate fertilizer and SMS (Na_2_SiO_3_·5H_2_O), a highly soluble Si source, examining its effects on root morphological traits, photosynthetic traits, and yield in soybeans [29,30,31,32]. These studies revealed that silicate fertilizers notably enhanced root traits, while SMS optimized photosynthetic traits [30]. Additionally, under salinity stress, there was a decrease in the expression of antioxidant genes and an increase in the expression of nitric oxide-related genes [29]. Furthermore, the study also found that Si increased the number of nodules in soybean, increased photosynthetic traits, and overall yield of the soybean [31]. However, there is still a lack of comparative studies on different forms of Si, particularly slow-release clay-based sources like Si zeolite. This creates a knowledge gap about how effective Si zeolite is during the early stages of rice development. One of the other reasons for comparing two forms of Si is their differences in solubility, chemical structure, availability to plants, and their capacity or speed of releasing plant-available Si. Previous research on zeolite has mainly focused on soil-grown crops, where it has been seen that the silica form of zeolite promotes crop growth and development. However, its role in soilless cultivation is less clear. While the effects of Si from SMS have been studied in both hydroponic and soil conditions, the effect of zeolite in soilless cultivation is less understood. Thus, this study aims to identify differences in phenotypic and physiological traits resulting from applying two Si forms under two cultivation methods. Specifically, this research examines the effects of two Si sources: Si zeolite and SMS, administered at different concentrations during the early seedling stage of rice grown in both hydroponics and soil.

## 2. Results

### 2.1. Results for EP I

#### Effect of Si on the Root Phenotypes

From the analysis of the variance (ANOVA) (Table 1), it was observed that all the root traits: total root length (TRL), surface area (SA), average diameter (AD), root volume (RV), the number of tips, and the fork numbers had a significant effect when treated with different concentrations and forms of Si. The level of significance was lower than 0.05 (*p* ≤ 0.05) for all the root traits. This signifies how the treatment of Si affected all the major root traits of rice seedlings. The replication, however, did not show a significant difference among the treatments except for TRL and the number of tips (*p* ≤ 0.05).

**Figure 1:**
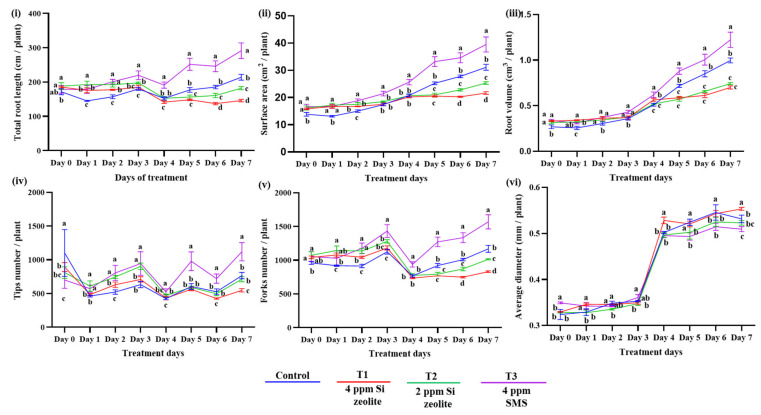
Effect of Si on (**i**) total root length, (**ii**) surface area, (**iii**) root volume, (**iv**) number of tips, (**v**) number of forks, and (**vi**) average diameter. The data are plotted as the mean value for each treatment. The different lowercase lettering indicates significant differences among the groups, while the same lettering indicates non-significant differences at a significance level of 0.05 (*p* ≤ 0.05). The letterings are based on Duncan’s Multiple Range Test (DMRT). The error bars indicate the standard error of the means within each treatment.

Although ANOVA revealed significant differences among treatments, only 4 ppm SMS Si showed higher root traits than the control at the end. Si from zeolite performed poorly compared to SMS Si, except for AD. Notably, by the fifth day, the gap in root traits widened. On the final day, 4 ppm SMS Si increased TRL by 36% over control. Zeolite at 4 ppm and 2 ppm reduced TRL by 31.7% and 15.0%, respectively (Figure 1i). A similar pattern was observed for SA; 4 ppm SMS Si increased SA significantly, while zeolite treatments showed modest or no increase. Day 7 saw a 34.2% SA increase for 4 ppm SMS Si, with zeolite treatments 29.0% and 13.3% lower (Figure 1ii). RV, tips, and forks were higher only with 4 ppm SMS Si at the end, with zeolite forms performing poorly. Final day, 4 ppm SMS Si increased RV, tips, and forks by 22.6%, 46.2%, and 34.2%, respectively, over control (Figure 1iii–v). Zeolite treatments had significantly lower values. AD showed a different pattern; 4 ppm zeolite had the highest AD (+4.1%), while 4 ppm SMS Si had the lowest AD at 0.51 mm, 4.2% below control (Figure 1vi).

### 2.2. Results for EP II

#### 2.2.1. Effects of Si on the Root Phenotypes

ANOVA analysis (Table 2) showed that Si treatments had a highly significant effect (*p* ≤ 0.05) on all measured root traits, including TRL, AD, RV, number of tips, SA, and number of forks. These results indicate that Si application significantly enhanced root traits related to both size (TRL, AD, RV, SA) and branching (tips, forks). In contrast, replication effects were not significant (*p* > 0.05) for any trait, suggesting that experimental replicates were consistent and contributed minimally to the overall variation.

**Figure 2:**
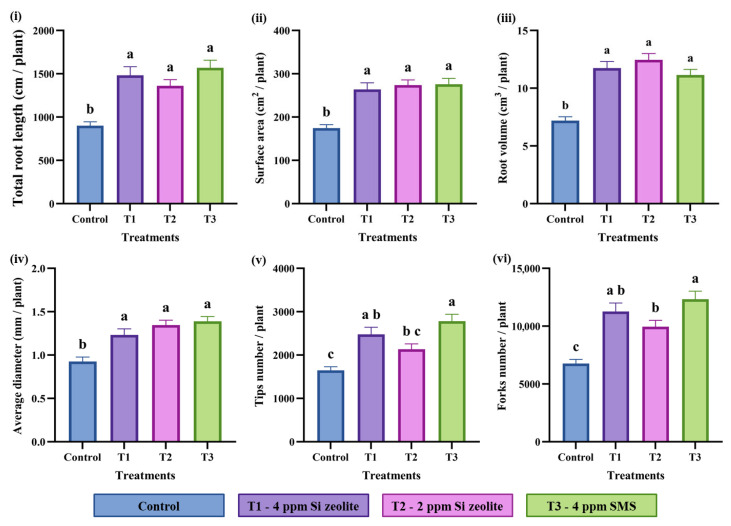
Effect of Si on (**i**) total root length, (**ii**) surface area, (**iii**) root volume, (**iv**) average diameter, (**v**) number of tips, and (**vi**) number of forks. The data are plotted as the mean value for each treatment. The different lowercase lettering indicates significant differences among the treatment groups, while the same lettering indicates non-significant differences among the treatment groups at a significance level of 0.05 (*p* ≤ 0.05). The letterings are based on Duncan’s Multiple Range Test (DMRT). The error bars indicate the standard error of the means within each treatment.

The effect of Si on rice seedlings in EP II, shown in Figure 2, revealed that all Si concentrations significantly (*p* ≤ 0.05) increased key root traits (TRL, RV, SA, AD, tips, and fork number). Compared to controls, 4 ppm Si zeolite (T1), 2 ppm Si zeolite (T2), and 4 ppm Si from SMS (T3) raised TRL by 62%, 50%, and 73%, respectively (Figure 2i). Similar increases were seen in SA (51%, 56%, 58%) (Figure 2ii), RV (32%, 45%, 50%) (Figure 2iii), with AD increasing by 63%, 72%, and 54% (Figure 2iv). Si-treated plants also had more tips—2100 to 2800—compared to 1600 in the controls, with increases of 50% (T1), 29% (T2), and 68% (T3) (Figure 2v). Fork numbers also rose over 40% with Si treatments (Figure 2vi), indicating Si boosts root elongation, radial growth, volumetric capacity, and branching number.

#### 2.2.2. Effect of Si on Seedling Dry Weight and Tiller Numbers

From the ANOVA analysis, it was observed that the Si treatment had a significant effect on the plant’s dry weight and the number of tillers per plant (Table 3). Root dry weight was strongly influenced by Si treatments (F = 9.1, *p* ≤ 0.05), while shoot dry weight showed a moderate but significant response (F = 3.91, *p* = 0.01). Similarly, the number of tillers were also significantly affected by the application of Si (F = 4.23, *p* = 0.0066). Replication effects were not significant (*p* > 0.05), confirming the reliability and reproducibility of the experimental design.

**Figure 3:**
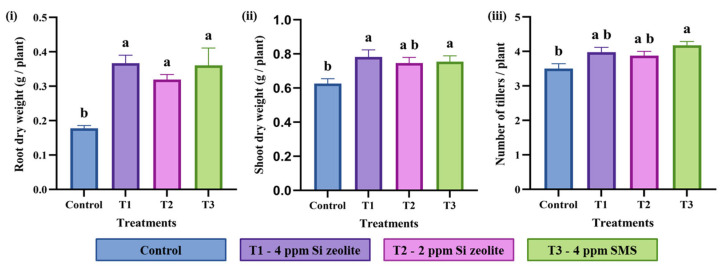
Effect of Si on dry weights and tiller numbers. Effect of Si on (**i**) root dry weight, (**ii**) shoot dry weight, and (**iii**) the number of tillers. The data are plotted as the mean value for each treatment. The different lowercase lettering indicates significant differences among the treatment groups, while the same lettering indicates non-significant differences among the treatment groups at a significance level of 0.05 (*p* ≤ 0.05). The letterings are based on Duncan’s Multiple Range Test (DMRT). The error bars indicate the standard error of the means within each treatment.

The effect of Si application on shoot and root dry weight of rice seedlings is shown in Figure 3. Si application also strongly affected root dry weight. The Si-treated plants had root dry weight between 0.32 and 0.37 g, while the control plants averaged about 0.18 g (Figure 3i). This indicates increases of 106% in T1 (4 ppm Si zeolite), 79% in T2 (2 ppm Si zeolite), and 103% in T3 (4 ppm Si from SMS) compared with the control. For shoot dry weight, Si fertilizer increased the values by approximately 24% in T1, 19% in T2, and 20% in T3 relative to the control (Figure 3ii). T1 and T3 treatments produced the highest increases. Similarly, the number of tillers was significantly affected by Si application. A notable increase in tiller number was seen in seedlings treated with pure Si (T3) compared to the control, with an increase of about 19% (Figure 3iii). Although the other two zeolite treatments did not show significant increases, they increased tiller numbers by 11%. These results demonstrate that Si application boosts both shoot and root biomass in rice seedlings and also increases the number of tillers per plant.

#### 2.2.3. Effect of Si on Photosynthetic Traits and Vegetative Indices

Table 4 presents the results of the ANOVA analysis for the photosynthetic traits and vegetative indices. In the case of photosynthetic traits, all measured parameters; electron transport rate (ETR), total conductance to water vapors (GTW), stomatal conductance to water vapor (GSW), and apparent transpiration (E-apparent) were highly significant (*p* ≤ 0.05), indicating that Si application had a significant influence on photosynthetic performance. Intensity (treatment days) also showed a significant effect (*p* ≤ 0.05) on all traits, demonstrating its strong influence on photosynthetic responses. Moreover, the interaction between treatment days and treatment was also significant (*p* ≤ 0.05) for all parameters, indicating that the effect of treatments varied according to the days of Si treatment.

In the case of VIs, the number of treatment days (intensity) had a significant effect (*p* ≤ 0.05), as did the interaction between intensity and treatment (*p* ≤ 0.05) in NDVI. However, treatment alone was not significant (*p* = 0.142). Similarly, PRI also showed no significant treatment effect (*p* = 0.5487), whereas the number of treatment days and the interaction term were highly significant (*p* ≤ 0.05). Overall, these results indicate that treatment alone did not significantly influence any of the VIs. Instead, variation was mainly caused by treatment days and their interaction with Si treatment.

**Figure 4:**
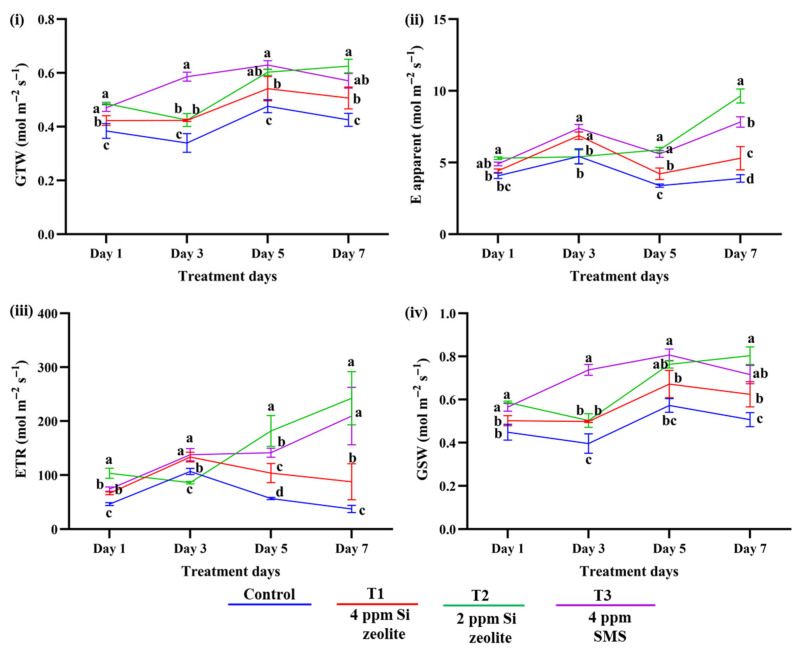
Effect of Si (**i**) total conductance to water (GTW), (**ii**) apparent transpiration (E-apparent), (**iii**) electron transport rate (ETR), and (**iv**) stomatal conductance to water vapor (GSW). The data are plotted as the mean value for each treatment. The different lowercase lettering indicates significant differences among the treatment groups, while the same lettering indicates non-significant differences among the treatment groups at a significance level of 0.05 (*p* ≤ 0.05). The letterings are based on Duncan’s Multiple Range Test (DMRT). The error bars indicate the standard error of the means within each treatment.

According to Figure 4, the applications of Si improved photosynthetic characteristics, as determined by the LI-600, throughout a seven-day period. All treatments showed significant improvements in GSW, GTW, E-apparent, and ETR when compared to the control. T2 (2 ppm Si zeolite) showed the greatest reaction on day 7, increasing GTW by 47.12% (Figure 4i), E-apparent by 147.72% (Figure 4ii), ETR by 5 times (Figure 4iii), and GSW by 58.52% (Figure 4iv) compared to the control. While T1 (4 ppm Si zeolite) exhibited notable gains across all metrics, T3 (4 ppm Si from SMS) also showed significant improvements, especially for E-apparent (101.11%) and ETR (460.97%). All things considered, these findings show that Si supplementation improved photosynthetic efficiency, with T2 (2 ppm Si zeolite) being the most effective treatment.

**Figure 5:**
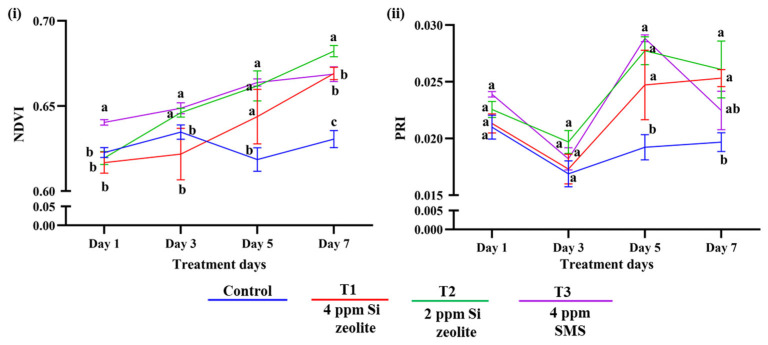
Effect of Si on VIs. Effects on the (**i**) normalized vegetative index (NDVI), and (**ii**) photochemical reflectance index (PRI). The data are plotted as the mean value for each treatment. The different lowercase lettering indicates significant differences among the treatment groups, while the same lettering indicates non-significant differences among the treatment groups at a significance level of 0.05 (*p* ≤ 0.05). The letterings are based on Duncan’s Multiple Range Test (DMRT). The error bars indicate the standard error of the means within each treatment.

Likewise, the VIs and their interaction with Si and the number of treatment days had a significant impact. In comparison to the control, the NDVI increased by 6.1% in T1 (4 ppm Si zeolite), 8.2% in T2 (2 ppm Si zeolite), and 6.0% in T3 (4 ppm Si from SMS) on day 7 of treatment (Figure 5i). This significant increase in NDVI was observed from the 5th day of treatment. With increases of 28.7%, 32.6%, and 14.2% in T1, T2, and T3, respectively, PRI showed a significant improvement, indicating increased pigment performance (Figure 5ii). Similar to NDVI, a significant increase in PRI between control and treatment was observed from the 5th day of treatment.

#### 2.2.4. Correlation Analysis Among the Indices

**Figure 6:**
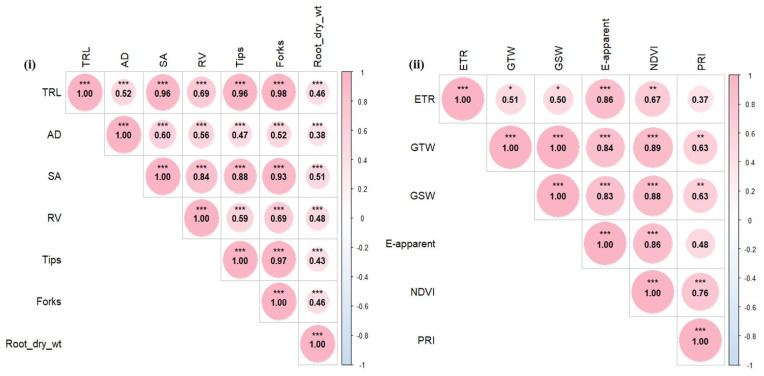
Correlation analysis for (**i**) root traits and (**ii**) photosynthetic and vegetative indices traits. * significant correlation at the 0.05 level; ** significant correlation at the 0.01 level, *** significant correlation at the 0.001 level. The legend bar indicates the values assigned to each color or correlation values (from 1 to −1).

Pearson’s correlation analysis was performed to explore the linear relationships among various traits in the second experiment. The root phenotypic traits showed significant positive correlations with each other (Figure 6i), while the final day’s data for photosynthesis and VIs traits were also positively correlated (Figure 6ii). Specifically, all root traits were significantly and positively correlated (Figure 6i), with the strongest correlations observed for TRL against SA, the number of tips, and the number of forks (all greater than 0.95). The number of tips also showed a high correlation with the number of forks. Similar patterns appeared in the photosynthetic traits and VIs, as all photosynthetic parameters; ETR, GTW, E-apparent, and GSW—were significantly correlated with both VIs, NDVI, and PRI (Figure 6ii). Notably, NDVI had a higher correlation coefficient with photosynthetic traits than PRI, indicating that increased NDVI, which reflects greener color or chlorophyll content, is associated with enhanced plant photosynthesis.

## 3. Discussion

The Si application is recognized as an essential approach to improve crop growth and stress resilience in cereals, especially rice [33]. This study explored how two forms of Si; zeolite and SMS impact the phenotypic and physiological traits during the early growth stages of rice under different growing conditions (hydroponics and soil). Thus, the study was conducted in two separate experiments (EP I and EP II). In EP I, where the rice seedlings were grown in a hydroponic solution, only 4 ppm of Si from SMS had significantly higher root traits (TRL, SA, RV, number of tips and forks) at the end of the experiment compared to the control. The other two treatments (Si zeolite at 4 ppm and 2 ppm) did not have a significant effect on the root traits, except for the AD, compared to the control. However, these zeolite forms of Si significantly increased all root traits when applied to soil-grown rice seedlings (EP II). To qualitatively illustrate the results, pictures of the rice plants taken during the study are shown in Supplementary Figure S1. From Figure S1, it is also evident that Si application significantly enhanced the morphological traits of the rice seedlings. Apart from the root traits, all Si treatments showed a significant increase in photosynthetic traits and the VIs of the rice seedlings. The number of tillers and the dry weight of the shoot and root were also significantly increased compared to the control group. To summarize the entire study, Si treatments applied in soil positively influenced root and shoot traits (EP II). In contrast, under hydroponic conditions, SMS enhanced most root parameters, whereas Si-zeolite exerted negative effects (EP I). Siliceous materials can strengthen plants, either directly by releasing soluble Si like silicic acid slowly from zeolites, or indirectly by improving soil properties [34]. Natural zeolites are crystalline aluminosilicates with a porous structure, and are widely used to improve poor soils and have high sorption and cation-exchange capacities [11,12,35,36]. Due to their high porosity and substantial cation-exchange capacity (CEC), natural zeolites exhibit excellent ion-exchange characteristics [37]. This high CEC allows zeolite to strongly adsorb nutrient cations (like NH_4_^+^, K^+^, Ca^+^, Mg^+^), which is beneficial in soils as a slow-release reservoir, but in hydroponics, it can temporarily remove nutrients from solution and delay their availability to plants, which can be interpreted as poor performance of zeolite-based media [38]. Because zeolites release Si slowly, they might be ineffective as short-term hydroponic fertilizers. In EP I, where rice was grown hydroponically for a week, rapid Si release from zeolite was unlikely; therefore, SMS (easily soluble in water) increased root growth, but Si-zeolite did not. Furthermore, there was a significant decrease in the root traits for Si zeolite applied treatments at the end of the experiment, which might be due to the high CEC of zeolites, which can adsorb essential nutrient cations from the hydroponic solution, thereby reducing nutrient availability and negatively affecting plant performance. However, in EP II, the interaction with microbes, pH, and organic acids in soil likely accelerated zeolite dissolution, making silicon available and leading to positive effects on plant growth. Furthermore, in terms of soil conditions, in some traits, the performance of Si from zeolite was better than that of Si from SMS. This might be due to the structure of the zeolite. The porous structure of the zeolite is known to reduce the nutrient leaching from the soil [39], and it is well known that sandy soils have a higher rate of nutrient leaching. Some research also demonstrated that the addition of zeolite increases water retention capacity in the root zones [40], and being slightly alkaline in nature, it helps to buffer the soil pH level [41]. Furthermore, the application of zeolites are proven to increase the soil organic matter content by 20.27% and significantly enhance the soil microbiomes as well [42].

In our study, EP I with 4 ppm of Si from SMS and all forms of Si in EP II exhibited significantly higher root traits, photosynthetic traits, VIs, dry weights, and tiller numbers. These positive effects of Si are not limited to visible morphological changes but are also closely associated with molecular and genetic processes inside the plant. Several studies have shown that Si regulates the expression of genes involved in root development, hormone signaling, and stress responses. For instance, the Si application can modulate auxin biosynthesis and transport pathways, which are essential for lateral root initiation and elongation, thereby promoting a more extensive and branched root system [32,43]. In rice, Si uptake and translocation are facilitated by specific transporter genes such as *Lsi1* and *Lsi2*, which enable efficient Si absorption from the soil and its movement within the plant [44]. The deposition of Si within plant tissues strengthens cell walls, enhances structural rigidity, and improves root penetration capacity, leading to greater water and nutrient uptake efficiency [45]. Moreover, Si has been reported to influence the expression of genes related to antioxidant defense and stress signaling, contributing to enhanced stress resilience and metabolic stability [43]. Furthermore, Si plays a significant role in regulating the expression of photosynthesis-related genes and maintaining photosynthetic performance under stress conditions. For example, Si treatment upregulated genes associated with photosystem I (*PsaH*) and photosystem II (*PsbY*), as well as those involved in electron transport (*PetC*, *PetH*) and light-harvesting complexes in rice exposed to heavy metal stress, thereby sustaining photosynthetic activity and improving resilience [46]. Similar physiological enhancements were observed by [47], who reported that Si supplementation improved gas exchange, chlorophyll retention, and water-use efficiency in *Solanum tuberosum* under drought stress—suggesting that Si influences both genetic regulation and physiological adaptation mechanisms. Collectively, these molecular and physiological processes demonstrate how Si enhances root system architecture, maintains photosynthetic efficiency, and promotes overall plant growth and stress tolerance by coordinating hormonal, structural, and gene expression responses.

Si has been demonstrated to improve plant development at the molecular level through controlling stress-responsive gene expression, antioxidant activity, and hormone signaling [17,43]. For instance, Tripathi et al. (2022) found that the application of Si increased the expression of genes involved in auxin production, such as YUCCA1 and TAA1 [32], which are directly related to root development [48]. Furthermore, Si deposition in plant tissues promotes effective CO_2_ uptake during photosynthesis, decreases oxidative damage, and increases structural rigidity [48,49]. Therefore, Si-mediated modulation of root gene expression and enhanced cellular metabolism may account for the improved physiological features seen in this study. These molecular processes offer a foundation for comprehending how Si improves rice root shape and shoot biomass [48,50]. Additionally, our findings demonstrate that Si-treated seedlings demonstrated more effective water and nutrient uptake, which is probably related to increased functional surface area and root branching [51]. Furthermore, compared to the control, Si application produced more tillers, suggesting that better root functionality promotes not only nutrient uptake but also increased tiller development and overall vegetative growth [51,52]. In rice, applying zeolite under flooded or alternate wetting–drying irrigation increases leaf chlorophyll (SPAD), plant dry matter, nitrogen accumulation, and grain yield [53]. Zeolite also promotes better root growth and nutrient uptake, which supports stronger above-ground growth and improved photosynthetic performance, including increased leaf area, higher chlorophyll content, and greater biomass [39]. Even under stress or variable water conditions, plants maintain better vegetative development due to enhanced water and nutrient dynamics in the soil–root system. Additionally, zeolite enhances soil potassium (K) availability and uptake, thereby improving stem morphology and yield stability in rice fields [54].

Upon close observation of the results from photosynthetic traits and the VIs (EP II), we found that significant differences in photosynthetic traits between the control and Si-treated groups appeared on the first day (GTW) and on the third day (E-apparent, ETR, and GSW) after treatment with Si. However, in VIs, the effect of Si was observed only on the fifth day after treatment. As photosynthetic traits are physiological traits related to parameters such as stomatal conductance and transpiration, they can be quickly affected by the plant’s metabolic processes. These traits can change within a short time in response to plant conditions and the environment. In natural environmental conditions, stomatal and photosynthetic activity change continually in response to changing environmental conditions [55]. However, VIs related to leaf pigmentation show slow change because it may take time to accumulate or reorganize pigmentation, and this change only occurs after a certain level of change in the physiological process has been reached. For example, to observe a significant increase in NDVI, the leaf should accumulate more green pigments, such as chlorophyll, which may take several days. The early observation of significant difference in photosynthetic traits has been supported by a study from Song et al., (2014) [46], where they found that the major photosynthesis-related genes like *Os08g02630* (*PsbY*), *Os05g48630* (*PsaH*), *Os07g37030* (*PetC*), and *Os03g57120* (*PetH*) increased significantly after adding 1.5 mM of Si in zinc-stressed rice plants at 72 h. This study also found Si increased the chlorophyll content in stressed rice.

Despite these promising results, the current study was limited to the early growth stage of rice. Although notable differences were observed within a week of Si treatment, their effects on later growth stages, yield components, and long-term productivity were not examined. Furthermore, the impact of Si on grain quality, stress adaptation, and the expression of specific genetic pathways remains unexplored in this study. Therefore, future research should aim to combine transcriptomic, proteomic, and metabolomic methods with large-scale field trials to better understand the molecular mechanisms and yield outcomes related to Si-induced improvements in rice. Expression levels of certain Si-related genes need to be explored under treatment conditions in both hydroponic and soil environments, allowing for a clearer understanding of the Si zeolite effect on hydroponics. Overall, future studies should focus on integrating phenotypic and genotypic studies to understand the correlation among these traits. The overall results indicate that the form of Si did not significantly influence the phenotypic and physiological traits under soil conditions. Both Si forms outperformed the control. Zeolite, as a slow-releasing source of Si and other essential nutrients, enhances soil fertility over time [56]. As previously discussed, it improves water retention, buffers soil pH, and increases organic matter and microbiome content. In contrast, Si derived from SMS is easily soluble, allowing plants to absorb it quickly and potentially give better performance within a short period of time. In terms of availability, zeolites are natural minerals found in specific locations, whereas SMS is a chemical compound that can be purchased. For short-term applications, SMS may be more cost-effective, but for long-term soil improvement, zeolite could offer better economic value. Therefore, based on these findings, the choice of the form of Si depends on the researcher’s or farmer’s goal. In hydroponics, the typical form of zeolite without any supplementation is not recommended. In such cases, a readily soluble form of Si, like SMS, can be a better option. In soil conditions, zeolites can be used if the goal is to improve long-term soil fertility or to address soil reclamation. For quick results, readily soluble SMS can be employed.

## 4. Materials and Methods

The study was divided into two separate experiments: Experiment 1 (EP I) and Experiment 2 (EP II), where EP I was followed by EP II. In EP I, the effect of different forms of Si treatments on root phenotypes was checked under hydroponic conditions in a non-destructive way, whereas in EP II, the effect of different forms of Si on root and shoot phenotypes was checked under soil growth conditions.

### 4.1. Plant Materials and Seedlings Establishment

Rice of the Ilphum variety was used in the experiment, as it is one of the popular rice varieties in Korea. The rice seeds were first sterilized with prochloraz fungicide (0.5 mL/L of water) for 24 h and then thoroughly washed with distilled water 4 to 5 times until the fungicide remnants were removed. For the EP I the seeds were grown in a growth chamber. The seeds were first grown in seedbed media (60% zeolite, 20% diatomite, 19.67% vermiculite, 0.3% fertilizer, and 0.03% of pH adjuster, Bunong Co., Ltd., Gyeongju-si, Gyeongbuk, Republic of Korea). When the seedlings reached the second vegetative growth stage, i.e., when the second true leaf emerged (V2 stage), the seedlings were transferred to the Hoagland’s solution contained in the hydroponic culture containers. The Hoagland’s solution was prepared by mixing the Hoagland salt medium and calcium nitrate in the ratio of 0.97 g and 0.66 g per liter of water (MB cell, YangJae-Dong, SeoCho-Gu, Seoul, Republic of Korea). The rice seedlings were fixed in the holes of the Styrofoam and secured with the help of upholstery foam. The seedlings were then grown for a week until the end of the treatment.

In the case of EP II, the seedlings were grown in outside environmental conditions. The sterilized rice seeds were first germinated in Petri dishes and then sown in polyvinyl chloride (PVC) pipes. The PVC pipes (40 cm in length and 10 cm in diameter) were filled with sandy soil, and the germinated seeds were carefully placed and covered with a thin layer of soil. The choice of sandy soil was made since it would be easy to harvest the roots without breaking the root parts in the sandy soil. Similarly, the sandy soil would make uniform growing conditions for the rice seedlings. Furthermore, sandy soils are known to be naturally less fertile, with lower organic matter content, and are less able to retain nutrients compared to other soil types [57]. Therefore, for normal growth and development of the plant, they were irrigated with a 1000-fold Hyponex solution (1 mL of Hyponex in 1 L of water). The Hyponex solution comprises essential plant nutrients, including nitrogen, phosphorus, and potassium, in a ratio of 7:10:6 percent. Irrigation was performed daily with 50 mL of Hyponex solution per pipe per day until the seedlings reached the second tillering stage.

### 4.2. Si Treatments and Experimental Design

In both experiments, the seedlings were treated with three different concentrations of Si along with a control group. The first two Si treatments were made using Eco Full Care (437, Immyeon-ro, Gokseong-gun, Jeollanam-do, Republic of Korea). This product contains a major ingredient, zeolite (30%), and water (70%). Zeolite is one of the naturally occurring Si minerals. The total content of soluble silicate in the Eco Full Care is 0.4%. For the first treatment, 1 mL of solution was diluted in 1 L of water. This corresponded to 4 ppm of Si. Similarly, for the second treatment, 0.5 mL of the Eco Full Care solution was diluted to 1 mL of water, corresponding to 2 ppm of Si. For the third treatment pure form of Si was used in the form of SMS. Initially, 0.4 g of Si was dissolved in 100 mL of water to create a stock solution containing 0.4% Si, matching the soluble Si content present in the Eco Full Care. After that, 1 mL of stock solution was diluted in 1 L of water, which corresponded to 4 ppm of Si. These concentrations of Si were chosen based on the results and conclusions from the previous studies on the effect of Si on plant growth under stressed or normal conditions [29,58,59]. The summary of the treatments used is given in Table 5.

In EP I, a single hydroponic container consisted of 15 rice seedlings representing a single replication for each treatment. Thus, a total of three replications, each with 15 plants per replication, were maintained throughout each treatment. The rice seedlings were treated with Si when they reached the V2 stage. The different concentrations of Si were poured into the hydroponic culture containers. As the zeolite was in liquid form in the Eco Full Care solution, it was poured directly into the nutrient solution after dilution. Furthermore, the nutrient solution was shaken with a glass rod at a regular interval so that the ions would not settle at the bottom. The roots were scanned daily until the seventh day of treatment. Similarly, in EP II, the rice seedlings were allowed to grow until the second tiller emerged. This was done to enable the measurement of more phenotypic and photosynthetic traits. As the rice leaves are small in diameter during the early growth stage (V2 stage), the photosynthesis and VIs measuring device could not capture the data; therefore, a later stage was chosen to obtain more phenotypic data. Each treatment was replicated four times, with 10 plants in each replication. When the rice reached the second tillering stage, they were treated with Si continuously for seven days. The photosynthetic and VIs data were monitored daily, whereas the roots and shoot traits were measured at the end of the experiment. Hyponex solution was continuously provided until the end of the experiment for all treatment groups, allowing only the effect of Si to be assessed.

### 4.3. Root Phenotypic Trait Measurement

The roots of the rice seedlings were scanned daily in the case of EP I, based on a non-destructive method of root analysis. The seedlings were removed from the solution and then placed in a transparent tray (30 cm × 40 cm) filled with clean tap water to ensure optimal visibility and spread. The tray was then kept on the scanner (Epson, 12000 XL, Nagano, Japan), and then the roots were scanned along with the shoots. These scanned images were of 4414 × 6156 pixels in size with a resolution of 400 dpi. The scanned root images were then analyzed using WinRHIZO Pro software (Regent Instruments Inc., Québec City, QC, Canada). Major root traits, including TRL, SA, AD, RV, number of tips, and number of forks, were obtained through the analysis. After each analysis, the seedlings were again kept in the Hoagland’s solution.

For EP II, the plants were harvested after a week of continuous Si treatments; thus, a destructive method of root analysis was performed. Each plant was carefully removed from the PVC pipes, and any adhering soil was gently washed off with clean water to prepare for root phenotyping. The cleaned root samples were placed into zip-lock plastic bags, with a small amount of fresh water added to prevent desiccation. These samples were then transported to the analytical laboratory for imaging and quantification of root traits. The same procedure for root image acquisition was performed as mentioned in EP I. Root traits similar to those mentioned in EP I were obtained through WinRHIZO analysis.

### 4.4. Photosynthetic Traits and Vegetative Indices Measurement

For measuring the photosynthetic traits, a LI-600 (LI-COR Inc., Lincoln, NE, USA) was used. The device was kept in Auto gsw + F mode while acquiring the data. The values of the parameters maintained during this mode are listed in Supplementary Table S1. The major photosynthetic traits measured were: ETR, GTW, GSW, and E-apparent. Similarly, the VIs were measured using PolyPen (RP410, Photon Systems Instruments, Brno, Czech Republic). The major VIs measured were NDVI, and PRI which provide non-destructive, rapid, and reliable insights into plant health, photosynthetic activity, and pigment dynamics. The measurements for the photosynthetic traits and the PolyPen were conducted in the morning, starting at 9 am. These traits were measured daily for all plants during the entire period of Si treatment. As mentioned earlier, the photosynthetic traits and the VIs were measured only in the second experiment, since EP II was conducted at a later stage of rice (when the second tiller emerged). This was done so that the leaves of the rice plant would be large enough to be clamped by the LI-600 and PolyPen.

### 4.5. Dry Weight and Number of Tillers Measurement

**Figure 7:**
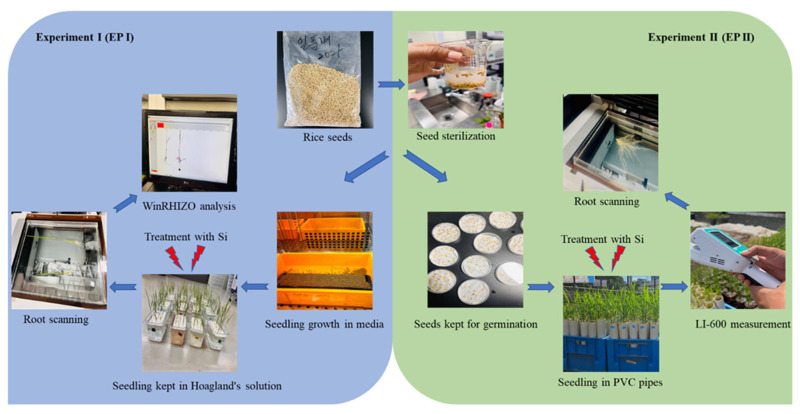
Workflow of the study, including the first and the second experiments.

At the end of the EP II experiment, the roots and shoots were cut and placed in separate envelopes after being patted dry. To ensure complete moisture removal, the envelopes were placed in a hot air oven (Model: JSON-150, Natural Convection Oven, Gongju City, Republic of Korea) at 60 °C for 72 h. Following the drying process, the root and shoot samples were weighed using an analytical balance, and their dry weights were recorded for further analysis. Similarly, to count the number of tillers, the harvested plants were first photographed using a Canon EOS 200 (Tokyo, Japan) with an image resolution of 72 dpi and an image size of 6000 × 4000. The number of tillers was then manually counted by observing the captured images. The overall workflow of the entire study (including EP I and EP II) has been illustrated in Figure 7.

### 4.6. Statistical Analysis

To evaluate the statistical significance of differences among treatments, a one-way analysis of variance (ANOVA) was performed using SAS software (version 9.4; SAS Institute Inc., Cary, NC, USA). The F-test was used to determine overall differences among group means, with significance thresholds set at *p* ≤ 0.05, *p* ≤ 0.01, and *p* ≤ 0.0001. When the ANOVA revealed significant effects, Duncan’s Multiple Range Test (DMRT) was applied to separate the means and identify specific differences between the control and Si treatment groups. Pearson’s correlation analysis was performed to illustrate the linear relationship between the traits in EP II. All graphical representations and data visualizations were generated using GraphPad Prism 10.6.1.

## 5. Conclusions

This study investigated the effect of different forms of Si (zeolite and SMS) in two different growing conditions of rice. In hydroponically grown rice (EP I), the SMS form of Si had a significant increment in the root traits compared to the control, whereas the zeolite form of Si had a significant negative effect compared to the control. Likewise, the soil-grown rice (EP II), both forms of Si, including all the treatment groups, showed a significant increase in every root trait (TRL, SA, RV, AD, number of tips and forks). In addition to root development, Si application improved photosynthetic parameters, VIs, root-shoot dry weight, and number of tillers, reflecting enhanced physiological performance and overall plant vigor. Overall, this study suggests that in hydroponic growing conditions, the zeolite form of Si is not ideal, and a readily soluble form of Si, like SMS, should be used. However, in soil conditions, the form of Si did not significantly matter, as all the Si treatments outperformed the control. Future research should aim to extend these findings to field conditions, later growth stages, and the integration of genomics and phenomics studies, as well as explore yield-related traits to better understand the long-term benefits of Si and its role in enhancing rice productivity and resilience under diverse environmental conditions.

## Figures and Tables

**Table 1:** Analysis of variance for the major root phenotypes for the first experiment (EP I).

Traits	Source	DF	Type III SS	Mean Square	F Value	Pr > F
TRL	Tre	3	690,146.406	230,048.8021	44.16	<0.0001
	Rep	2	39,626.1723	19,813.0861	3.8	0.0242
	Error	174	906,483.503	5209.6753		
RV	Tre	3	6.45307648	2.15102549	33.37	<0.0001
	Rep	2	0.12150165	0.06075083	0.94	0.3916
	Error	174	11.2159784	0.06445965		
AD	Tre	3	0.05959097	0.01986366	6.8	0.0002
	Rep	2	0.00396271	0.00198135	0.68	0.5087
	Error	174	0.50807265	0.00291996		
Tips	Tre	3	8,767,979.63	2,922,659.88	20	<0.0001
	Rep	2	2,520,019.31	1,260,009.65	8.62	0.0003
	Error	174	25,282,230.1	146,140.06		
Forks	Tre	3	18,660,454.3	6,220,151.44	32.37	<0.0001
	Rep	2	796,286.88	398,143.44	2.07	0.129
	Error	174	33,432,739.8	192,142.18		
SA	Tre	3	10,691.8502	3563.95007	59.85	<0.0001
	Rep	2	345.0447	172.52235	2.9	0.0578
	Error	174	10,361.3085	59.54775		

Note: TRL—Total root length, SA—surface area, RV—root volume, and AD—average diameter.

**Table 2:** Analysis of variance for the major root phenotypes of the second experiment (EPII).

Traits	Source	DF	Type III SS	Mean Square	F Value	Pr > F
Length	Treatment	3	10,397,967.3	3,465,989.1	13.92	<0.0001
	Replication	3	744,366.87	248,122.29	1	0.3961
AD	Treatment	3	5.24376431	1.74792144	12.45	<0.0001
	Replication	3	0.11579006	0.03859669	0.27	0.8435
RV	Treatment	3	666.742904	222.247635	22.29	<0.0001
	Replication	3	32.5281679	10.8427226	1.09	0.3562
Tips	Treatment	3	28,386,725.9	9,462,241.98	12.45	<0.0001
	Replication	3	1,612,419.36	537,473.12	0.71	0.5491
SA	Treatment	3	283,129.051	94,376.3502	14.66	<0.0001
	Replication	3	21,945.1376	7315.0459	1.14	0.3363
Forks	Treatment	3	696,596,338	232,198,779	15.58	<0.0001
	Replication	3	35,920,130.2	11,973,376.7	0.8	0.4937

Note: TRL—Total root length, SA—surface area, RV—root volume, and AD—average diameter.

**Table 3:** Analysis of variance for dry weight and number of tillers of the second experiment (EP II).

Traits	Source	DF	Type III SS	Mean Square	F Value	Pr > F
Root dry weight	Treatment	3	0.93364094	0.31121365	9.1	<0.0001
	Replication	3	0.02191286	0.00730429	0.21	0.8869
Shoot dry weight	Treatment	3	0.57344606	0.19114869	3.91	0.01
	Replication	3	0.06291333	0.02097111	0.43	0.7323
Tiller number	Treatment	3	9.01242	3.00414	4.23	0.0066
	Replication	3	0.93101	0.31034	0.44	0.7268

**Table 4:** Analysis of the variance of photosynthetic traits and vegetative indices of the second experiment (EP II).

Traits	Source	DF	Type III SS	Mean Square	F Value	Pr > F
ETR	Treatment	3	375,813.828	125,271.276	11.85	<0.0001
	Replication	3	76,235.56	25,411.853	2.4	0.072
	Trt days	7	613,617.15	87,659.593	14.75	<0.0001
	Trt days × Trt	21	877,765.004	41,798.334	7.03	<0.0001
	Error	100	1,057,290.785	10,572.908		
GTW	Treatment	3	1.13995168	0.37998389	26.37	<0.0001
	Replication	3	0.08283356	0.02761119	1.92	0.1318
	Trt days	7	613,617.15	87,659.593	14.75	<0.0001
	Trt days × Trt	21	877,765.004	41,798.334	7.03	<0.0001
	Error	100	1.44075466	0.01440755		
E-apparent	Treatment	3	337.249913	112.416638	72.71	<0.0001
	Replication	3	31.0774833	10.3591611	6.7	0.0004
	Trt days	7	960.584067	137.226295	126.19	<0.0001
	Trt days × Trt	21	856.4905664	40.7852651	37.5	<0.0001
	Error	100	154.6144675	1.5461447		
GSW	Treatment	3	2.2432758	0.7477586	24.28	<0.0001
	Replication	3	0.19021098	0.06340366	2.06	0.1105
	Trt days	7	2.0805672	0.29722389	40.84	<0.0001
	Trt days × Trt	21	0.81260932	0.03869568	5.32	<0.0001
	Error	100	3.07918007	0.0307918		
NDVI	Treatment	3	0.0236345	0.0078782	1.84	0.142
	Replication	3	0.02271317	0.00757106	1.77	0.1554
	Trt days	7	0.33190381	0.04741483	21.39	<0.0001
	Trt days × Trt	21	0.14013621	0.00667315	3.01	<0.0001
	Error	152	0.75597914	0.00497355		
PRI	Treatment	3	0.00030608	0.00010203	0.71	0.5487
	Replication	3	0.00086383	0.00028794	2	0.1167
	Trt days	7	0.00866467	0.00123781	17.01	<0.0001
	Trt days × Trt	21	0.00962351	0.00045826	6.3	<0.0001
	Error	152	0.02190846	0.00014413		

Note: ETR—Electron transport rate, GTW—total conductance to water vapor, GSW—stomatal conductance to water vapor, E-apparent—Apparent transpiration, NDVI—normalized difference vegetative index, PRI—photochemical reflectance index, Trt days—number of days of treatment, Trt—treatment, and “×” indicates the interaction between the sources.

**Table 5:** Description and concentration of the silicon treatments used in the study.

SN	Treatments	Si Concentration	Description
1	Control	Not applicable	Normal water in EP I and Hyponex solution in EP II
2	T1	4 ppm of Si from zeolite	1 mL of Eco Full Care in 1 L of water
3	T2	2 ppm of Si from zeolite	0.5 mL of Eco Full Care in 1 L of water
4	T3	4 ppm of Si from SMS	1 mL of Si stock solution in 1 L of water

## Data Availability

The data and results are presented within the study. Further inquiries can be directed to the corresponding author.

## Supplementary Materials

The following supporting information can be downloaded at: https://www.mdpi.com/article/10.3390/plants15020176/s1. Figure S1: Setup of the experiment and rice seedlings under each treatment for the first experiment (EP I). Figure S2: Setup of the experiment and rice seedlings under each treatment for the second experiment (EP II). Table S1: LI-600 parameters kept during the data acquisition process.

## Abbreviations

The following abbreviations are used in this manuscript:

Si	Silicon
RGB	Red, green, and blue
VIs	Vegetative indices
GSW	Stomatal conductance to water vapor
GTW	Total conductance to water vapors
ETR	Electron transport rate
E-apparent	Apparent transpiration
NDVI	Normalized difference vegetative index
PRI	Photochemical reflectance index
TRL	Total root length
SA	Surface area
RV	Root volume
AD	Average diameter
SMS	Sodium metasilicate
