# Supplementary material for: Effect of Silicon on Early Root and Shoot Phenotypes of Rice in Hydroponic and Soil Systems
Source: Plants (Basel). 2026 Jan 6;15(2):176. doi: 10.3390/plants15020176 (PMC12845091; doi:10.3390/plants15020176)
Supplement: Supplementary file 1 [file plants-15-00176-s001.zip › plants-4046204-supplementary.pdf]

Setup of the study (EP I)

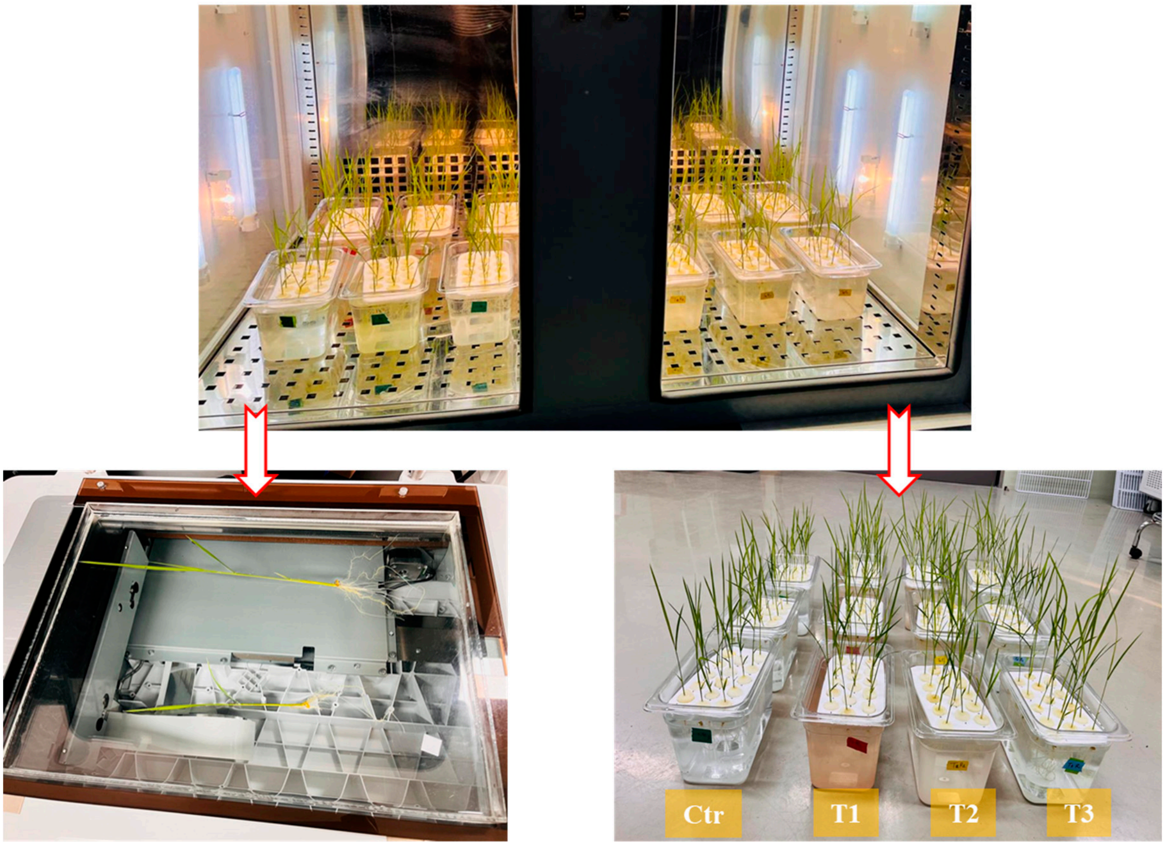

Scanning of the seedlings **Comparison of the seedlings**

Figure S1: Setup of the experiment and rice seedlings under each treatment for the first experiment (EP I).

Ctr: Control, T1: 4 ppm Si zeolite, T2: 2ppm Si zeolite, and T3: 4 ppm Si from sodium metasilicate.

Setup of the study (EP II)

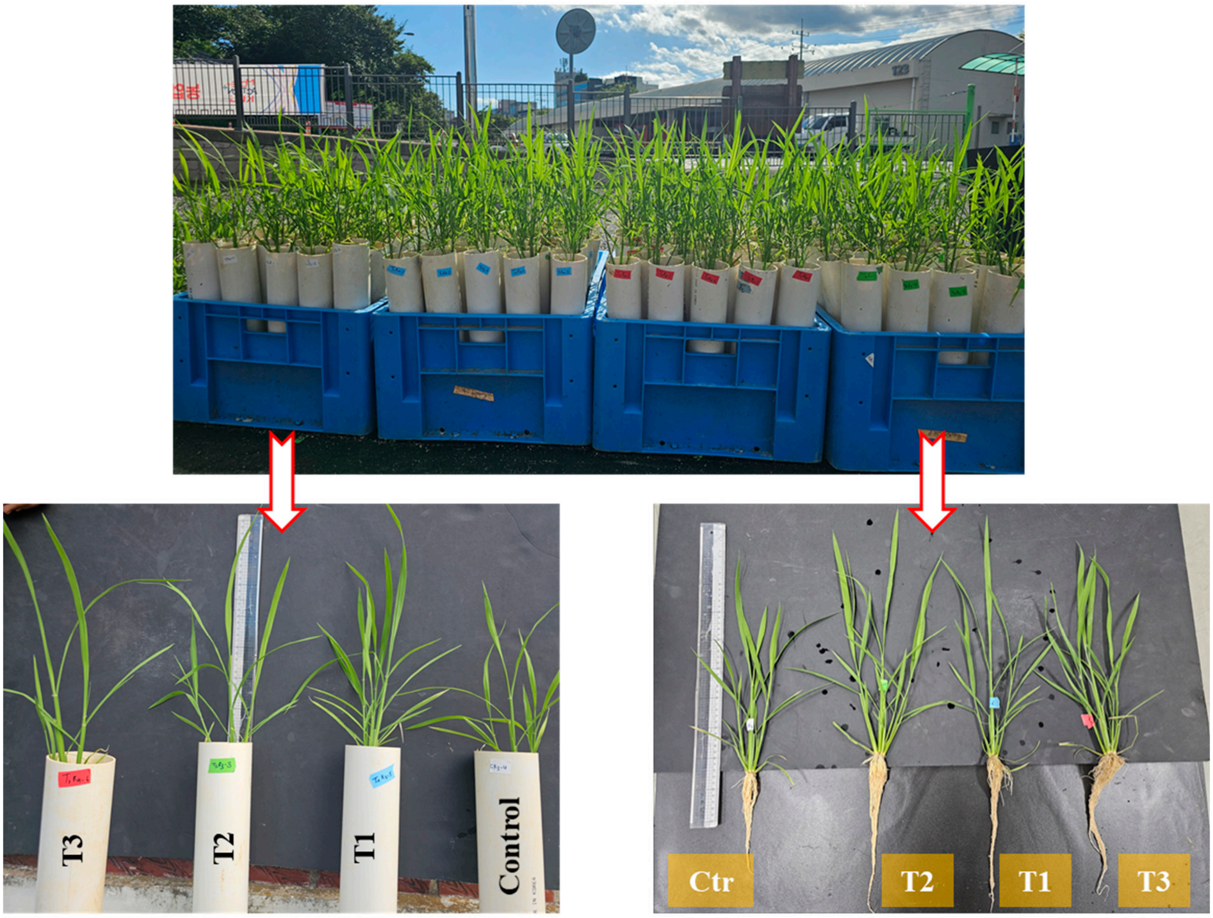

Comparison before harvest **Comparison after harvest**

Figure S2: Setup of the experiment and rice seedlings under each treatment for the second experiment (EP II).

Ctr: Control, T1: 4 ppm Si zeolite, T2: 2ppm Si zeolite, and T3: 4 ppm Si from sodium metasilicate.

Table S1. LI-600 parameters kept during the data acquisition process.

Parameters	Values
Flow	150
Match frequency	10
Dark adoption	No
Leaf absorptance (Leaf abs)	0.80
Absorptance PSII (Abs PS II)	0.5
Actinic modulation rate (Act mod rate)	500
Flash type	mpf
Intensity	7000
Phase	1/2/3:300/300/300
Ramp	25
